# Benzimidazole Derivatives as Energetic Materials: A Theoretical Study

**DOI:** 10.3390/ma14154112

**Published:** 2021-07-23

**Authors:** Jonas Sarlauskas, Jelena Tamuliene, Svajone Bekesiene, Alexander Kravcov

**Affiliations:** 1 Life Sciences Centre, Department of Xenobiotic Biochemistry, Institute of Biochemistry, Vilnius University, Sauletekio av. 7, 01513 Vilnius, Lithuania; jonas.sarlauskas@bchi.vu.lt; 2 Physics Faculty, Institute of Theoretical Physics and Astronomy, Vilnius University, Sauletekio av. 3, 01513 Vilnius, Lithuania; jelena.tamuliene@tfai.vu.lt; 3 Research Group on Logistics and Defence Technology Management, General Jonas Zemaitis Military Academy of Lithuania, Silo 5a, 10322 Vilnius, Lithuania; 4 Department of Construction Technology, Faculty of Civil Engineering, Czech Technical University in Prague, Thákurova 7/2077, Prague 6—Dejvice, 16629 Prague, Czech Republic

**Keywords:** benzimidazole nitro compounds, explosives, detonation pressure, detonation velocity, sensitivity, toxicity, substituents

## Abstract

The explosive properties and stability of benzimidazole compounds are studied to determine the influence of substituents and their position. The results obtained reveal the conjugation of substituents as one of the crucial factors for the thermal stability of these compounds. We also found that two -CH_3_ substituents increase the thermal stability of the parent compound, while nitro groups decrease it. Moreover, the study clearly exhibits that the combination of an -NO_2_ substituent with -CH_3_ does not change the stability of the benzimidazole. On the other hand, nitro groups increase the chemical stability and explosive properties of the compounds under investigation, but their sensitivity could not fully satisfy the requirements of their safety and increase their toxicity. The main results of the study indicate that high thermal and chemical stability, low toxicity and sensitivity, and good explosive properties could be achieved by the precise combination of nitro, -CH_3_, and triazole ring substituents. These findings are very important for the design of new, effective, and non-sensitive explosives.

## 1. Introduction

The synthesis of energetic materials with high density, detonation velocity, pressure, thermal and chemical stability has been a research focus in the past decade. Low costs and high yields in synthesis, low toxicity, high chemical and thermal stability, etc., are the main criteria that new energetic materials (explosives) should fulfill. These criteria are skeleton-molecule dependent and they are crucial in considering which substituents improve the explosive properties of materials and how they affect thermal stability. For example, the connection of two imidazole rings obtaining 2,2′-bisimidazoles follow the trend of generating larger energetic molecules, and the thermal stability of nitrobenzene is increased due to the introduction of amino groups ([1] and refs. cited herein). A decrease in the sensitivity of the product relative to the unsubstituted starting material is obtained due to the introduction of amino groups into the nitroaromatic molecules [2,3]. An increase in the number of methyl substituents enhances the thermal stability and reactivity of methylammonium perchlorates [4]. Moreover, when hydrogens at the 3- and 5-positions on the picric acid ring are substituted with two amino groups, the resulting compound—ammonium diamino picrate, i.e., picric acid substituted with amino groups—show enhanced thermal stability and shock insensitivity, marking it out as a powerful secondary explosive with improved practical characteristics [5]. The conjugation (three or more p orbitals joining together) also increases the stability of high energy materials and decreases their sensitivity. The energetic materials containing a triazole ring have relatively low molecular weight, high nitrogen content and density, good thermal stability, low impact sensitivity, and a large explosive volume, thus fulfilling the criteria for the use of such compounds as potential explosive materials [6].

Benzimidazole is poorly investigated with respect to their polynitro derivatives, although the study of Klapötke et al. exhibited 2-aminobenzimidazole as a valuable and inexpensive starting material for the synthesis of energetic materials [7]. Szala et al. described the new energetic compound 5,5′,6,6′-tetranitro-2,2′-bibenzimidazole as a secondary explosive with high thermal stability [8]. It is also necessary to mention the work of Politzer et al. and Pang et al. who presented studies on innovative energetic materials [9,10,11].

In this context, we performed this study with the aim of clarifying how various substituents and their position influence the stability and energetic properties of benzimidazole in order to determine if this could be used in the manufacture of explosive materials. Our goal is to offer new ways of producing eco-friendly, strong-brisance, and inexpensive explosives. This is the novelty of our research. Considering the fact that superior performance, stability, and eco-friendly qualities are important for the development of new explosives to be used in practice, we selected only a comparatively small, consolidated group of structurally related compounds. This was accomplished by excluding many other nitro benzimidazole derivatives, particularly those containing halogen, N_3_, hydrazine, and other unacceptable functional groups due to their potential role in increasing chemical reactivity, as hazards for health or the environment, and their inappropriate properties that affect chemical stability.

## 2. Methods of Investigation

The structure of the molecules of new explosive materials were studied via Becke’s three-parameter hybrid functional, applying the non-local correlation provided by Lee, Yang, and Parr (B3LYP) [12,13]. The structures of each studied molecule were modeled and optimized without any symmetry constraint. This means that at least three different structures of the same molecule were studied. The structures were different due to the location of substituents or the dihedral angles of the -NO_2_ groups with respect to the molecule skeleton. The lowest total energy was obtained, taking into account zero-point energy corrections. A comparison of the total energy of each molecule’s conformers allowed us to select the most stable one for further studies. The binding energy per atom—the energy required to decompose a molecule into its constituting atom—was evaluated to compare the thermal stability of the compounds investigated. It is calculated as follows:

(1) $$\mathrm{BEA}=\frac{E-\sum E_{N}}{N}$$

where E is the total energy of the molecule, E_N_ is the energy of the atom, N is the number of atoms constituting a molecule. This energy is related to the structural arrangement of molecules that could appear due to environmental temperature changes [14]. Hence, a higher BEA indicates higher resistance to the above-mentioned temperature increase.

The HOMO–LUMO gap, electronegativity, chemical hardness, and chemical softness were calculated to estimate chemical stability with the following equation:

(2) $$\chi =\frac{I+A}{2}$$

(3) $$\eta =\frac{I-A}{2}$$

(4) $$S=\frac{1}{2\eta }$$

where χ is the electronegativity, η is the chemical hardness, S is the chemical softness, I is the ionization energy, and A is the electron affinity calculated as the difference of total energies of neutral and ionized molecules [15,16]. Equation (5), presented by Türker et al. and used to assess detonation velocity [17], is as follows:

(5) $$D_{1}^{2}=-393.6877-0.2454\left(\frac{\mathrm{NE}}{M}\right)-114.0793\frac{E}{M}$$

where M is the molecular weight (in g/mol), N is the number of NO_2_ groups, E is the total energy (au) of the molecule under study.

When the detonation velocity is known, the detonation pressure could be obtained by the following equation:

P (kbar)=15.58 [D ρ/(1.01(1 + 1.30 ρ)]^2^ (6)

where D is detonation velocity in km/s, and ρ is density in g/cm^3^ as predicted by ACD Labs software ACD/ChemSketch (version 11.0, Advanced Chemistry Development, Inc., Toronto, Ontario, Canada) [18]. ACD/ChemSketch calculates the density from molecular weight and the calculated molar volume from additive increments.

The Kamlet–Jacob equations were used to evaluate detonation pressure with the following formula [19]:

(7) $$p=15.58\rho^{2}\times N\times {\left(M\times Q\right)}^{0.5}$$

where p is the detonation pressure in kilobar, N is the number of moles of gaseous detonation products per gram of explosive, M is the average weight of these gases in gram of gas per mole of gas, Q is the chemical energy of the detonation reaction in calories per gram, and ρ is the density of the undetonated explosive in gram per cubic centimeter.

Oxygen balance expresses the degree to which an explosive can be oxidized and indirectly provide information on the sensitivity, strength, and brisance of an explosive. Therefore, this parameter was calculated to compare the above-mentioned properties of the explosive investigated [20]. Oxygen balance calculations were performed by Equation (8):

(8) $$\mathrm{OB}=\frac{-1600}{M}\left(2a+\frac{b}{2}+Z-d\right)$$

where M is the molecular weight (g/mol), a is the number of atoms of carbon, b is the number of atoms of hydrogen, d is the number of atoms of oxygen, and Z is the number of atoms of metal (metallic oxide produced).

Additionally, the impact sensitivity was estimated by Equations (9) and (10), provided in [21]:

(9) $${\mathrm{logh}}_{1}=\frac{11.76a+61.72b+26.89c-11.48d}{M}$$

(10) $${\mathrm{logh}}_{2}=\frac{47.33a+23.50b+2.357c-1.105d}{M}$$

where M is the molecular weight (g/mol), a is the number of atoms of carbon, b is the number of atoms of hydrogen, c is the number of nitrogen atoms, d is the number of atoms of oxygen.

The model was used in our previous studies, along with experimental conditions [22]. The results obtained revealed a correlation among theoretical predictions and experimental results.

## 3. Results and Discussions

Several structures of seven molecules were modeled, and following the recommendations, each of the molecule structures was optimized. The analysis results for thermal and chemical stability as the binding energy per atom as well as the HOMO–LUMO gap are presented in Section 3.1. Moreover, the detonation velocity, which is one of the performances that enables the selection, tailoring, and understanding of the behavior of explosives in terms of expected effects, was established (see in Section 3.2). Consequently, the oxygen balance was determined to specify the shock sensitivity and predict the effect of substituents on it (see in Section 3.3). Additionally, a detailed study of bond length and angle changes due to the substituents is presented (see in Table A1, Table A2, Table A3, Table A4, Table A5, Table A6 and Table A7, Appendix A). For data modelling, the Gaussian program was used; for analysis results illustration, the Gauss-View package was applied [22,23].

### 3.1. Thermal and Chemical Stability

**Figure 1:**
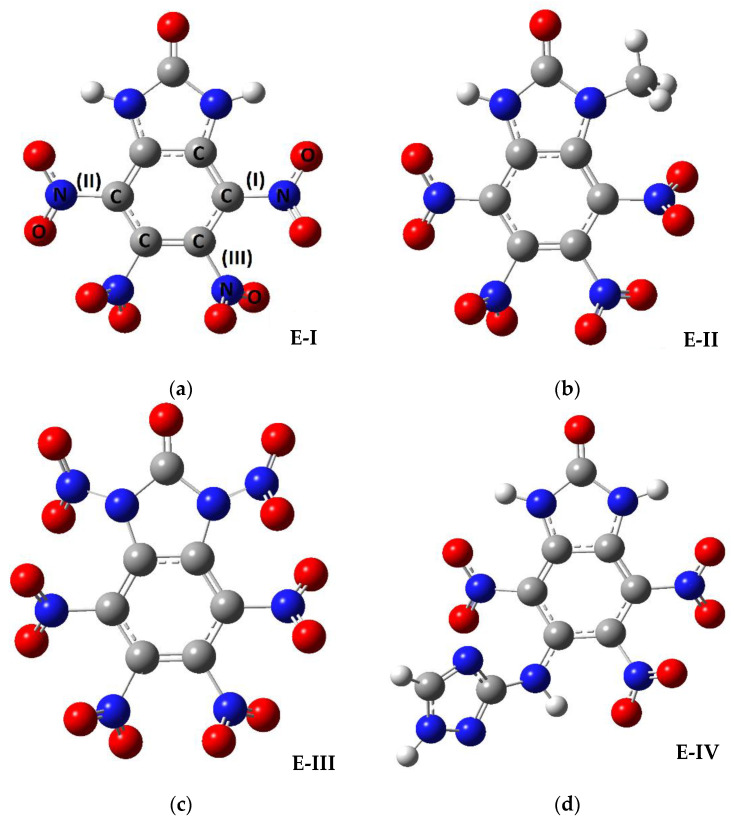

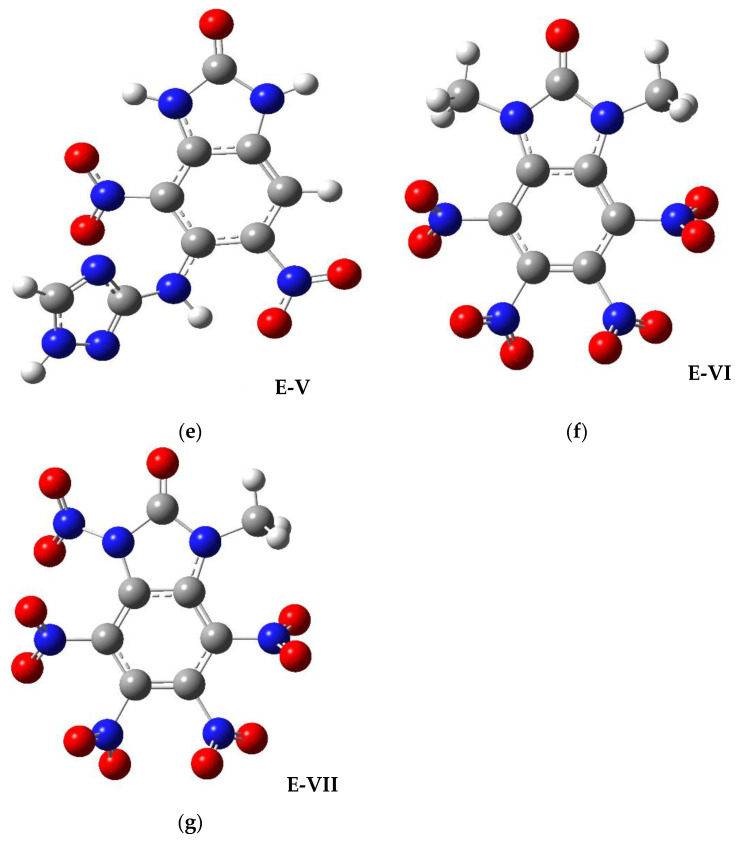
The structures of the molecules under investigation: (**a**)—initial molecule (E-I), (**b**)—the initial molecule with a methyl substituent joined to the imidazole ring (E-II), (**c**)—the initial molecule with two additional nitro groups joined to the imidazole ring (E-III), (**d**)—the initial molecule with a C_2_H_3_N_4_ substituent to the benzene ring (E-IV), (**e**)—the initial molecule with a C_2_H_3_N_4_ (triazolyl amino) ring and 7-H to the benzene ring (E-V), (**f**)—the initial molecule with two methyl groups joined to the imidazole ring (E-VI), (**g**)—the initial molecule with nitro and methyl substituents joined to the imidazole ring (E-VII). The abbreviation of the molecule names is used here and further in the text.

It is necessary to mention that the main difference between the molecules investigated is the substituents and their position. The parent molecule consists of four nitro groups, imidazol-2-one, and benzene rings. The substituents in the imidazole or benzene rings are the following: NO_2_, CH_3_, H, and C_2_H_3_N_4_. The molecular structures are shown in Figure 1.

It is necessary to mention that the compounds investigated are non-planar. The difference between the shortest and the longest CC bonds remains approximately the same in all compounds presented. They exhibit a uniform conjugation effect in the benzene ring and imply that in the case of E-I, E-II, E-III, E-VI, and E-VII, the stability and explosive properties are changed only due to substituents, while in the case of E-IV and E-V, these changes could occur due to additional conjugation in the imidazole ring. 

In this paper, we did not present a detailed study of bond length and angle changes due to the substituents (these data are presented in Table A1, Table A2, Table A3, Table A4, Table A5, Table A6 and Table A7, Appendix A). The main geometric structure change is clearly shown in Figure 1, and the nearest nitro group to the substituent rotating with respect to its position in the initial molecule due to the steric effect is presented in Table 1.

It is necessary to mention that the compounds investigated are non-planar. The difference between the shortest and the longest CC bonds remains approximately the same in all compounds presented. They exhibit a uniform conjugation effect in the benzene ring and imply that in the case of E-I, E-II, E-III, E-VI, and E-VII, the stability and explosive properties are changed only due to substituents, while in the case of E-IV and E-V, these changes could occur due to additional conjugation in the imidazole ring.

These additional imidazol-2-one substituents, and consequently, the geometric structure changes, lead to the variableness of the thermal and chemical stability. Indeed, a comparison of the binding energy per atom (BEA) indicates that E-IV and E-V with ‘additional conjugation’ are the most thermally stable compounds among those investigated, i.e., their BEA is the largest. However, the influence of -CH_3_ and nitro substituents on the thermal stability depends on their combination. This influence can be described as follows: two nitro groups decrease the stability of the parent compound; -CH_3_ and -NO_2_ substituents do not significantly change this stability; two methyl groups increased this stability. This dependence is related to different N-C (305 kJ/mol) and N-N (160 kJ/mol) bond energies. When a bond is strong, i.e., there is higher bond energy, more energy is necessary to break this bond. Hence, the thermal stability could be increased through conjugation and the formation of stronger chemical bonds.

It is interesting that ‘additional conjugation’ decreases the chemical stability of the germ (initial compounds), while -CH_3_ and -NO_2_ substituents in the imidazole ring of these compounds increase it. The gap values of HOMO–LUMO are shown in Table 2.

These results present a comparison of the chemical hardness obtained and confirm that ‘additional conjugation’ could lead to faster aging in comparison with the addition of -CH_3_ and -NO_2_ groups to the imidazole ring.

It is necessary to emphasize that a higher (in some cases significantly) thermal stability of the new explosive material in comparison to that of TNT and tetryl was obtained (Table 2).

The values of hardness, presented in Table 2, indicate the high chemical stability of E-III. The chemical stability of E-V is the lowest, taking into consideration the smallest value of chemical hardness of the other compounds. We predict that the decrease in the thermal stability of these compounds is related to the decrease in the energy of their bonds; that is, an N-H bond, whose energy is equal to 391 kcal, is changed by a C-N or N-N bond, whose energy is 305 or 109 kcal [24]. Moreover, the comparison of BEA demonstrates that the conjugation of substituents is the crucial factor in the increase of thermal stability of the compounds investigated.

These data are not enough to allow us to draw conclusions on chemical stability because the analysis described above only enable us to understand the reactivities of the compound, i.e., to clarify which compounds tend to undergo a chemical reaction. Hence, the maximum hardness index Y was calculated, as follows:

Υ = 1 − 2S^2^ (11)

where S is chemical softness. It can be seen that hard-hard binding frame is preferred in molecules where Υ holds values over 0.5 [25]. The chemical bond can still be formed as the soft-soft combination in the molecules where Υ stands below 0.5 values; however, this is only possible with positive non-zero figures. Only negative values of Υ indicate an anti-bonding character that can further be associated with anti-binding entropy. The result of the analysis of the maximum hardness index indicates that all compounds investigated are chemically stable.

We thus state that nitro substituents increase the toxicity of materials. This follows from the comparison of electronegativity values where a larger value indicates a higher chemical activity, i.e., the possibility to create new chemical bonds with other materials and change their properties. These values are large in the compounds possessing an additional nitro group. For example, the electronegativity, and consequently, the toxicity of E-III and E-IV are larger than those of E-I and E-V, respectively. Moreover, the smallest electronegativity values of E-II, E-IV, E-V, and E-VI presented in Table 2 also indicate that -CH_3_ and triazolyl amino substituents decrease the toxicity of these explosives.

### 3.2. Detonation Velocity and Pressure

Detonation velocity is one of the performances that enable the selection, tailoring, and understanding of the behavior of explosives in terms of expected effects. A lot of strategies that consider a variety of input parameters are used to obtain this particular parameter. However, the Kamlet–Jacobs method is still considered more reliable than any contemporary or new method for the prediction of detonation velocity. We would like to emphasize that the density of the material and the chemical energy of detonation are usually included in well-known approaches such as those of L.R. Rothstein [26], Xiong et al. [27], and J.R. Stine [28].

The evaluation of the density and energy of detonation is a very difficult task from a theoretical point of view. First, there are no evident rules to recognize the products of the decomposition reactions of explosives. The Kistiakowsky–Wilson rules should only be used for explosives with an oxygen balance greater than −40 [20], and the usage of the suggested overall stoichiometry for an explosive with the general formula of C_a_H_b_N_c_O_d_ leads to an altered decomposition of the explosive, and as a consequence, different detonation energy [28,29]. Moreover, there are also several ways to evaluate the energy of detonation [29,30,31,32]. Second, the effective volume of the molecule in crystal could not be correctly estimated, and the quantity of interaction index does not adequately indicate the potential for intermolecular interactions [33].

Lemi Türker [17] suggests equations for density of velocity calculated, which include the total energy and the number of NO_2_ groups presented in molecules. These relations are developed by regression analysis—statistical curve fitting for selected explosives to find the coefficients that were used.

Considering the facts presented above, we calculated the detonation velocity by using the equation suggested by Türker along with the Kamlet–Jacobs (K–J) equation:

(12) $$D_{2}=1.01{\left({\nu M}^{1/2}Q^{1/2}\right)}^{1/2}\left(1+1.30\rho \right)$$

where D is the detonation velocity in km/s, Q is density in g/cm^3^, ν is the moles of gaseous detonation products per gram of explosive (in mol/g), M is the average molecular weight of gaseous products in g/mol, Q is the chemical energy of detonation in kJ/g. The results obtained are presented in Table 3.

The results presented indicate that the detonation velocity for the same compounds investigated corresponds fairly well. A good matching of results obtained through the Kamlet–Jacobs (K-J) and Türker equations is observed. Hence, it is possible to predict the detonation velocity quite well without any knowledge of material density, the chemical energy, and the final product of the detonation. Hence, the approaches used are sufficiently correct to obtain detonation velocity and pressure, and are capable of producing qualitative results fairly well. In any case, only additional nitro groups can significantly improve explosive properties (power).

It is known that detonation velocity is an important property to be taken into consideration when rating an explosive. The detonation velocities for high explosives range from 3300 fps to 29,900 fps (1.01 km/s to 9.11 km/s). Hence, the results obtained indicate that the compounds under investigation are highly explosive.

The detonation pressure calculated prove the statement above (Table 4).

It is necessary to mention the detonation pressure of the TNT molecule, which is equal to 213–259 kbar and is used as a standard. The detonation pressure of E-V is only lower than that of TNT. Referring to the results obtained, we could classify the materials as high brisance (with the exception of E-V) and state that additional nitro substituents increase detonation pressure, while those of -CH_3_ and triazole decrease it. Hence, these results lead to the conclusion that the nitro group increases the ability of the molecules under investigation to do the work, which does not depend on the position of the nitro group in the molecule.

### 3.3. Sensitivity

The oxygen balance is determined to indicate the shock sensitivity and foresee how it is influenced by the substituents (Table 4). Likewise, the similar parameters of TNT and tetryl were calculated for comparison. The analysis of the oxygen balance indicates that E-IV, E-VI, and E-V are less sensitive, and shows the relative sensitivity sequence of these molecules: (1) E-V; (2) TNT; (3) E-IV; (4) E-VI; (5) tetryl; (6) E-II; (7) E-I; (8) E-VII; (9) E-III. Hence, additional -NO_2_ groups increase the shock sensitivity of E-I, while the triazole ring decreases it. A comparison of the oxygen balance of the E-IV and E-V compounds proves this observation.

The results presented in Table 5 indicate that the sensitivity of E-IV and E-V is comparable with that of tetryl and TNT, respectively. The -CH_3_ substituent decreases the sensitivity of primary molecules, but this decrease is not as effective as it is in the case of the triazole ring. The sensitivity of E-V is higher than that of TNT and tetryl.

Hence, the results obtained show that nitro substituents improve the chemical stability and explosive properties of the materials under investigation, but their thermal stability and sensitivity could not satisfy the requirements of safety. On the other hand, the thermal stability and insensitivity of the primary molecule increase due to the triazole ring and the amino substituents, although the chemical stability and explosive properties become worse than those of the primary molecule. These results are important for the design of the new explosives.

## 4. Conclusions

Benzimidazole compounds were investigated, with the aim of observing the influence of various substituents and their positions on the compounds’ stability. The explosive properties were also studied to reveal the most important features for the modeling of high-energy materials.

The results obtained indicate that the conjugation of the substituents is the crucial factor for thermal stability. The compounds under study with a conjugated imidazole ring could decompose in higher temperatures as compared to other compounds in the study. We also observed that the thermal stability of the initial compound could be increased when substituents such as two -CH_3_ were used. However, although nitro groups used as substituents could decrease stability, its combination with -CH_3_ does not change the stability of the benzimidazole derivative. We also found that multiple nitro substituents can increase the toxicity of the compounds.

On the other hand, the nitro group substituents were found to increase the chemical stability and explosives properties of the compounds under investigation, but their sensitivity could not fully satisfy the requirements of safety.

This summary of the main results have led us to the conclusion that high thermal and chemical stability, low toxicity and sensitivity, and good explosive properties can be achieved precisely by combining nitro, -CH_3_, and triazole ring substituents. These findings are important for the design of high-energy materials in the selected benzimidazole class.

## Figures and Tables

**Table 1:** Selected bond length (A) and dihedral angles (degrees) of the molecules under investigation.

Abbreviation of Material	C-N, (I)	C-N, (II)	C-N, (III)	O-N-C-C, (I)	O-N-C-C, (II)	O-N-C-C, (III)
E-I	1.47	1.47	1.49	22.26	19.33	64.51
E-II	1.48	1.47	1.48	64.243	18.963	47.07
E-III	1.48	1.48	1.49	46.45	48.051	59.64
E-IV		1.45	1.45		23.16	8.79
E-V	1.47	1.46	1.46	33.13	24.24	32.92
E-VI	1.48	1.48	1.48	63.2	118.1	51.6
E-VII	1.48	1.48	1.49	48.7	118.0	59.5

Source: authors’ calculations.

**Table 2:** Binding energy per atom (BEA) and the HOMO–LUMO gap of the compounds under investigation.

Abbreviation of Material	BEA, eV	HOMO–LUMO Gap, eV	Chemical Hardness, eV	Chemical Softness, eV	Electronegativity, eV	Y, Hardness Index
E-I	5.697	3.493	1.746	0.286	6.259	0.84
E-II	5.562	3.696	1.848	0.271	5.959	0.85
E-III	5.523	4.251	2.126	0.235	6.703	0.89
E-IV	6.089	3.314	1.450	0.345	5.267	0.76
E-V	6.160	3.049	1.525	0.328	4.746	0.78
E-VI	5.843	3.984	1.992	0.251	5.656	0.87
E-VII	5.684	4.079	2.040	0.245	6.186	0.88
TNT	5.376	5.023	2.512	0.199	6.231	0.92
Tetryl	5.262	4.307	2.154	0.232	6.217	0.89

Source: authors’ calculations.

**Table 3:** The detonation velocity obtained.

Abbreviation of Material	D_1_ km/s	D_2_ km/s	Deviation, %
E-I	8.64	8.15	5.67
E-II	8.28	7.79	5.92
E-III	9.46	9.46	0.0
E-IV	7.26	7.32	0.83
E-V	6.53	6.53	0.00
E-VI	7.93	8.18	3.06
E-VII	9.03	9.87	8.5

Source: authors’ calculations.

**Table 4:** Detonation pressure of the compounds investigated.

Abbreviation of Material	Density, g/cm^3^	Detonation Pressure, When D = D_1_,kbar	Detonation Pressure, When D = D_2_,kbar
E-I	1.860	338.00	300.71
E-II	1.780	302.02	267.21
E-III	2.11	447.75	435.54
E-IV	1.85	195.20	241.58
E-V	1.73	176.26	184.45
E-VI	1.72	271.33	289.00
E-VII	1.92	448.87	375.26

Source: authors’ calculations.

**Table 5:** Oxygen balance and sensitivity.

Abbreviation of Material	Oxygen Balance	Logh1	Logh2
E-I	−30.56	1.50	1.22
E-II	−43.88	1.85	1.45
E-III	−3.96	1.11	0.83
E-IV	−61.50	1.84	1.59
E-V	−83.61	2.15	1.90
E-VI	−55.14	1.87	1.64
E-VII	−27.43	1.36	1.20
TNT	−73.97	2.38	1.98
Tetryl	−47.37	2.15	1.57

Source: authors’ calculations.

## Data Availability

Data sharing not applicable.

## Appendix A

**Table A1:** Convergent geometries of E-I.

Atom	X, Å	Y, Å	Z, Å
C	0.01321	−0.01916	−0.01145
C	0.01317	0.01911	1.41142
C	1.20539	0.02087	2.11021
C	2.41349	0.01547	1.388
C	2.41355	−0.01537	0.01207
C	1.20546	−0.02076	−0.71021
N	1.16519	−0.0802	3.57188
N	3.71836	0.11118	2.09869
N	3.71855	−0.1115	−0.69835
N	1.16506	0.08057	−2.17187
N	−1.29013	0.02341	1.80464
C	−2.15379	−0.00016	0.69991
N	−1.29006	−0.02353	−0.40476
H	−1.59628	−0.115	−1.36003
H	−1.59642	0.11496	2.75988
O	−3.35163	−0.00015	0.69987
O	0.11583	−0.25792	−2.71182
O	2.14017	0.52155	−2.74193
O	2.14068	−0.52051	4.14181
O	0.11589	0.25787	4.11195
O	3.91652	−1.14863	−1.2933
O	4.46601	0.83708	−0.59031
O	4.46604	−0.83713	1.98982
O	3.91635	1.14811	2.69398

**Table A2:** Convergent geometries of E-II.

Atom	X, Å	Y, Å	Z, Å
C	0.99434	−0.06911	−0.06212
C	0.36888	1.20024	−0.04709
C	−1.01354	1.27497	0.00952
C	−1.76389	0.1005	0.01485
C	−1.14701	−1.13548	−0.02513
C	0.24426	−1.22887	−0.076
N	−1.69529	2.58718	0.03905
N	−3.24542	0.1914	0.05776
N	−1.97133	−2.3728	−0.03384
N	0.89683	−2.55304	−0.17223
N	1.34524	2.1657	−0.14116
C	2.61161	1.57974	−0.17462
N	2.35611	0.16956	−0.17065
N	3.39722	−0.72566	0.35315
C	1.20585	3.61656	−0.25057
O	3.67031	2.13101	−0.19064
O	1.73326	−2.6631	−1.04968
O	0.54796	−3.40048	0.61919
O	−2.40668	2.85529	−0.90904
O	−1.46577	3.28972	1.0045
O	−1.83853	−3.09315	−1.00028
O	−2.69765	−2.53978	0.9225
O	−3.85116	−0.43987	−0.78217
O	−3.71181	0.89911	0.92792
O	4.47875	−0.57283	−0.12618
O	3.02284	−1.48904	1.20851
H	2.1959	4.00806	−0.46232
H	0.83132	4.04109	0.67659
H	0.53399	3.86826	−1.0689

**Table A3:** Convergent geometries of E-III.

Atom	X, Å	Y, Å	Z, Å
N	−2.00772	−1.09821	0.08383
C	−0.67606	−0.69663	0.03377
C	−0.66314	0.70825	−0.03296
N	−1.98723	1.13433	−0.08114
C	−2.88391	0.02625	0.00198
C	0.53545	1.39489	−0.11081
C	1.72595	0.6722	−0.06184
C	1.71333	−0.70469	0.05934
C	0.50976	−1.40528	0.10997
N	0.55913	2.86514	−0.2759
O	−0.19045	3.30745	−1.12687
N	3.0293	1.38424	−0.16951
O	3.20183	2.01694	−1.18876
N	3.00346	−1.44067	0.16518
O	3.76485	−1.32246	−0.76995
N	0.50652	−2.87572	0.27505
O	1.24346	−3.51425	−0.44195
O	−4.06804	0.03718	0.0028
O	1.30862	3.48996	0.44017
O	3.78971	1.25197	0.76454
O	3.16576	−2.07639	1.18422
O	−0.2498	−3.30412	1.12717
N	−2.50016	−2.39042	−0.46178
N	−2.45499	2.43549	0.46488
O	−3.50826	2.79883	0.04699
O	−1.70823	2.936	1.26498
O	−3.55937	−2.73437	−0.04247
O	−1.76391	−2.90441	−1.26307

**Table A4:** Convergent geometries of E-IV.

Atom	X, Å	Y, Å	Z, Å
N	0.018077	0.177191	0.032973
C	0.034242	0.210705	1.393189
C	1.397925	0.099761	1.782690
N	2.133972	−0.014586	0.632181
C	1.314379	0.056451	−0.493928
C	1.715219	−0.001509	3.112232
C	0.701177	0.144467	4.083560
C	−0.660920	0.252475	3.728974
C	−0.978758	0.201107	2.335411
N	3.084519	−0.402408	3.455362
O	3.969650	−0.029620	2.690663
N	1.108079	0.415827	5.456916
O	0.396779	−0.012328	6.366497
N	−2.336196	−0.009903	1.841945
O	−3.136342	−0.575056	2.561451
O	1.634149	0.030098	−1.650990
O	3.242412	−1.121701	4.419452
O	2.113308	1.083084	5.625729
O	−2.567547	0.342254	0.686362
H	−0.816248	0.266234	−0.523941
H	3.139810	−0.020742	0.597439
N	−1.611272	0.449794	4.693170
C	−2.715320	1.280465	4.610094
N	−3.618651	1.291071	5.573963
N	−4.464143	2.257821	5.138473
C	−4.045217	2.756239	3.966795
N	−2.926651	2.163239	3.600779
H	−5.275596	2.495494	5.682467
H	−4.563365	3.528900	3.424514
H	−1.341047	0.165678	5.625834

**Table A5:** Convergent geometries of E-V.

Atom	X, Å	Y, Å	Z, Å
N	0.015274	0.115231	0.024863
C	0.016879	0.117281	1.386016
C	1.350532	−0.100953	1.796558
N	2.104253	−0.218502	0.638547
C	1.308225	−0.081212	−0.489970
C	1.664789	−0.209807	3.118764
C	0.649481	−0.036424	4.072491
C	−0.705663	0.228005	3.715624
C	−1.009149	0.215526	2.321149
H	2.666471	−0.403044	3.466805
N	1.088071	−0.103592	5.454480
O	2.290594	−0.136023	5.679604
N	−2.368279	0.133524	1.810684
O	−3.235310	−0.343649	2.520314
O	1.635387	−0.115741	−1.648575
O	0.241203	−0.142280	6.357997
O	−2.552031	0.492698	0.646654
H	−0.800096	0.287293	−0.540108
H	3.095893	−0.363849	0.576068
N	−1.634188	0.504392	4.675115
C	−2.704988	1.376301	4.586740
N	−3.619108	1.404500	5.541249
N	−4.411744	2.422440	5.126009
C	−3.955211	2.933092	3.972626
N	−2.862852	2.298741	3.601705
H	−5.219376	2.680607	5.666005
H	−4.430460	3.744260	3.447406
H	−1.336333	0.259349	5.613734

**Table A6:** Convergent geometries of E-VI.

Atom	X, Å	Y, Å	Z, Å
C	−1.095515	−0.713123	−0.003624
C	−1.096864	0.710949	0.003389
C	0.103007	1.399533	0.000074
C	1.306373	0.692320	0.008124
C	1.307687	−0.689682	−0.008398
C	0.105818	−1.399331	−0.000251
N	0.117404	2.877685	0.020515
N	2.583742	1.441866	0.049516
N	2.586555	−1.436958	−0.049881
N	0.123528	−2.877381	−0.019732
N	−2.405356	1.117327	0.031651
C	−3.244845	−0.003084	−0.000127
N	−2.403154	−1.121987	−0.031864
C	−2.957491	−2.465967	−0.150673
C	−2.962137	2.460362	0.149718
O	−4.449547	−0.004310	−0.000169
O	−0.371623	−3.436022	0.940856
O	0.611651	−3.407449	−0.999059
O	0.601834	3.408057	1.001498
O	−0.376846	3.435862	−0.940755
O	2.725659	−2.311300	0.782996
O	3.372952	−1.119858	−0.918362
O	3.372889	1.123074	0.914937
O	2.719358	2.319680	−0.780353
H	−4.030018	2.338855	0.303764
H	−2.781698	3.038273	−0.752603
H	−2.529095	2.975756	1.005218
H	−2.522074	−2.980724	−1.005340
H	−4.025306	−2.346236	−0.306579
H	−2.777651	−3.043508	0.752005

**Table A7:** Convergent geometries of E-VII.

Atom	X, Å	Y, Å	Z, Å
C	0.994388	−0.069040	−0.062212
C	0.368909	1.200263	−0.047152
C	−1.013517	1.274975	0.009428
C	−1.763822	0.100505	0.014838
C	−1.146904	−1.135496	−0.025011
C	0.244355	−1.228832	−0.075885
N	−1.695247	2.587185	0.038901
N	−3.245361	0.191374	0.057615
N	−1.971174	−2.372819	−0.033467
N	0.897005	−2.552978	−0.171760
N	1.345243	2.165765	−0.141203
C	2.611613	1.579847	−0.174682
N	2.356151	0.169619	−0.170973
N	3.397209	−0.725623	0.352902
C	1.205735	3.616619	−0.250357
O	3.670281	2.131165	−0.190682
O	1.733195	−2.663352	−1.049365
O	0.547001	−3.400703	0.618848
O	−2.406152	2.855501	−0.909498
O	−1.465575	3.289834	1.004228
O	−1.838585	−3.093224	−0.999884
O	−2.697448	−2.539635	0.922938
O	−3.851007	−0.440419	−0.781985
O	−3.711862	0.899176	0.927638
O	4.479083	−0.571799	−0.125296
O	3.022285	−1.490391	1.206784
H	2.195750	4.008257	−0.462037
H	0.831192	4.040964	0.676881
H	0.533862	3.868425	−1.068636
